# High occurrence of cyclosporiasis in Istanbul, Turkey, during a dry and warm summer

**DOI:** 10.1186/1756-3305-3-39

**Published:** 2010-04-23

**Authors:** Melda Ozdamar, Elif Hakko, Salih Turkoglu

**Affiliations:** 1 Department of Clinical Microbiology, Anadolu Medical Center, Kocaeli 41400, Turkey; 2 Department of Infectious Diseases, Anadolu Medical Center, Kocaeli 41400, Turkey; 3 Department of Microbiology and Clinical Microbiology, University of Istanbul, Istanbul Medical Faculty, 34093, Capa, Istanbul, Turkey

## Abstract

We evaluated the incidence of *Cyclospora cayetanensis *in immunocompetent, diarrheic patients during the summers of 2006-2009 in Istanbul. Stools from 1876 patients were examined using microscopic techniques. *Cyclospora *oocysts were observed in wet preparations by light and epifluorescence microscopy and in fecal smears that were stained by Kinyoun's modified acid-fast stain. Characteristic *Cyclospora *oocysts were observed in 2 patients in 2006, 17 in 2007, and one in 2009. Samples positive for *Cyclospora *were further analyzed by a single step polymerase chain reaction (PCR) with *Cyclospora*-specific primers from the ITS-1 region of the genome.

The majority of the *Cyclospora *positive cases (15) were clustered during about 15 days in June 2007, indicating an unusual incidence of cyclosporiasis in this time period. The climatic characteristics of 2007 could have played a role in this high occurrence rate.

## Findings

*Cyclospora cayetanensis *is a frequently reported emerging pathogen that causes diarrhea in humans. It was first described as causing prolonged, watery diarrhea in humans in Papua New Guinea by Ashford in 1979. The species designation *Cyclospora cayetanensis *was given in 1994 to Peruvian isolates of human-associated *Cyclospora *by Ortega and colleagues [1]. This pathogen is found worldwide but predominates in tropical and subtropical areas [2]. After ingestion and infection in the host, the parasites' unsporulated oocysts are excreted with the feces and must sporulate to be infectious. This requires 7 to 15 days under a certain temperature (23 to 27°C) and humidity [3]. Outbreaks of diarrheal disease in humans have been linked to the consumption of raspberries, salads, basil and Asian freshwater clams; have been reported in North, Central and South America, Asia, Africa, Australia, England, and Eastern Europe [3-9]. These outbreaks are usually associated with importation of contaminated food products or contaminated water sources [2,9]. *Cyclospora cayetanensis *is primarily regarded as a cause of traveler's diarrhea, and it is not usually included in the routine laboratory algorithms for immunocompetent patients, unless there is a specific reason such as prolonged diarrhea or a history of travel to an endemic region. Although several case reports indicate the presence of *Cyclospora cayetanensis *disease in different regions of Turkey, epidemiological data on this pathogen are lacking [10-12]. In our microbiology laboratory in a 209 beds new hospital in Istanbul, we included *Cyclospora *testing in our routine algorithm for diarrheal disease diagnostics. We monitored the incidence *Cyclospora *for four consecutive seasons which peaked in the summer of 2007. In analyzing the cases, we found a high occurrence of cyclosporiasis which could be related to the unusual climatic conditions that occurred during 2007.

A total of 1876 patients (31% pediatric, 69% adult) with diarrhea were admitted to the Anadolu Medical Center outpatient clinics during June, July, and August of 2006, 2007, 2008, and 2009.

Stools were routinely investigated for coccidian parasites in the microbiology laboratory in the summer months. Direct wet mount examination was followed by formalin-acetate concentration (Para Pak, Meridian Diagnostics), Kinyoun's modified acid-fast stain. The oocysts were mostly diagnosed during a wet mount microscopic examination. Further or definitive diagnosis was made using stained smears. Characteristic oocysts appeared as spherical structures of 8-10 μm in diameter, with variable acid-fast staining: some stain dark red or pink, and some remain unstained. As a standard test, autofluorescence under ultraviolet (UV) fluorescence illumination was also used for the diagnosis of *Cyclospora *infection in stool samples.

Samples found to be C*yclospora*-positive upon microscopic examination were subjected to DNA extraction. Briefly, 100 μL of stool suspension was vortexed vigorously with acid washed glass beads (SIGMA Glass beads, 425-600 μm) for 10 minutes in the lysis buffer provided in the DNeasy Blood & Tissue Kit (QIAGEN Inc. California), and DNA was purified according to the manufacturer's instructions. A single-step polymerase chain reaction (PCR) with *Cyclospora*-specific primers from the ITS-1 (first internal transcribed spacer) region of the *Cyclospora *genome (Ccits37f-*GCTTGCTATGTTTTAGCATGTGG *and Ccits501r-*GCACAATGAATGCACACACA*) was performed using 10 μL of purified DNA [13].

**Figure 1:**
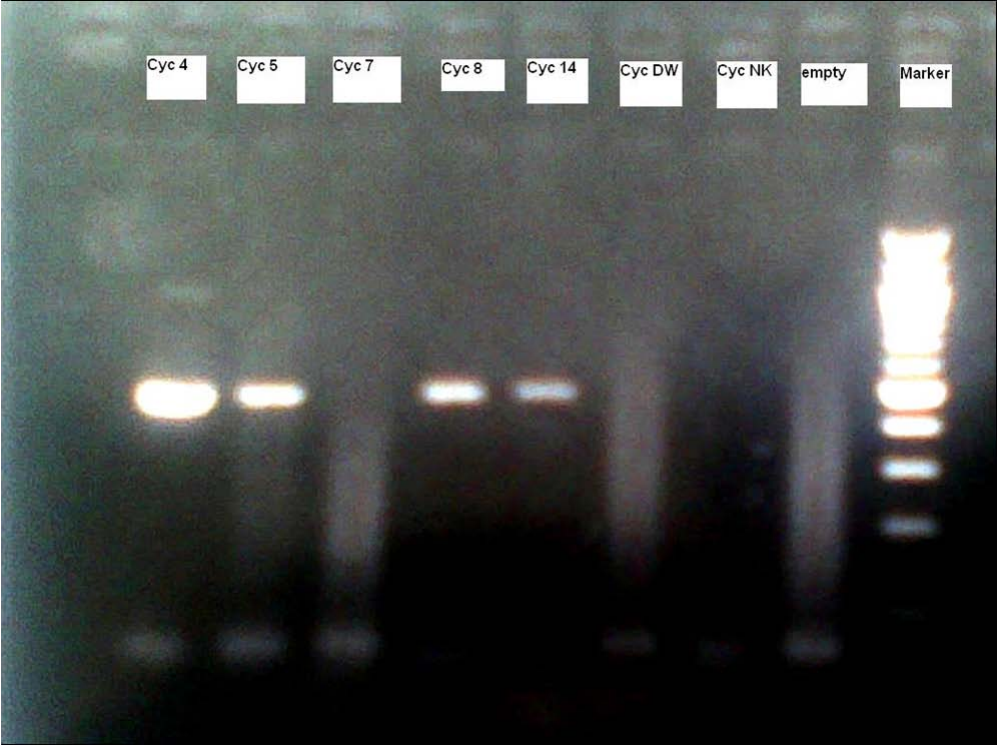
**Agarose gel electrophoresis showing *Cyclospora *PCR results**. Positive = 456 bp band.

A total of 17 cases of cyclosporiasis were detected among 505 stool samples from patients with diarrhea in 2007, and 14 samples were positive for *C. cayetanensis *by PCR (Figure 1). Only 2 cases were detected in 506 stool samples from patients with diarrhea (with both microscopic examination and PCR) in 2006, no cases were detected in 465 samples in 2008, and one case were detected in 400 stool samples over the same period in 2009. The majority of the cases were detected in a 15-day time period in 2007.

Stool cultures for *Salmonella *and *Shigella*, *Aeromonas *species, and *Rotavirus *and *Adenovirus *antigens gave negative results in these C*yclospora*-positive samples. Fifty stool samples belonging to 2006-2009 from asymptomatic persons were screened for C*yclospora *oocysts and found to be negative.

Diarrhea was persistent in most of our patients, with half of them also presenting with fever in 2007. All of the patients were successfully treated with trimethoprim-sulfamethoxazole (TMP/SMZ 160/800 mg/bid). After 7-10 days, all patients were negative for *Cyclospora *oocysts upon stool examination.

According to patient records, 41% (7/17) of the cases reported were connected with additional cases, including colleagues and family members who described similar symptoms. We believe that the total number of patients affected by C*yclospora *infection was higher than the 17 cases reported. All of the patients were from a high socioeconomic background, and had a good sanitary condition. The number of patients admitted to the outpatient clinics of the hospital is about 3,500 per month, and the total number of diarrhea cases in the 6^th^, 7 and 8^th ^months is about 500 to 600. There was no evident elevation in the total number of diarrhea cases in the month of July in the summer of 2007. How did a number of people of relatively high socioeconomic status surprisingly contract this pathogen over a very limited time period?

There have only been a limited number of cases of cyclosporiasis detected in Turkey thus far, and these were mainly imported cases from the warmer southern parts of the country and in poorer cities or villages with inferior infrastructure [10-12]. Studies of the prevalence of *Cyclospora cayetanensis *are limited, and as in other similar parts of the world the lack of epidemiological data is primarily due to unsatisfactory worldwide performance in the detection of a divergent multitude of diarrheal pathogens. The diagnostic algorithms are out-of-date, and do not include this pathogen. There are no positive data on *Cyclospora *cases in the Ministry of Health of Turkey records for 2007. Taking the *Cyclospora *prevalence from 2006 to 2009 as a baseline (there is no country-wide baseline level of endemicity), is the high prevalence in 2007 worth addressing?

There are some observations that could explain this increased prevalence in 2007.

1) There was no snowfall in the previous winter, which is not normal, in addition to the very high overall temperature in the summer months. 2) Importantly, the water levels of Istanbul drinking water reservoirs during 2007 were nearly empty, and water usage was very limited, which have resulted in insufficient food washing for items such as fruits and vegetables, especially those that cannot be peeled (e.g., lettuce and raspberries). 3) The city water is regularly screened for coccidia by the Istanbul Water and Sewerage Administration, and the results were negative from 2006-2009. 4) All of the C*yclospora*-positive patients were admitted over a period of 15 days, and 88.2% (15/17) had a prolonged (7-15 days) history diarrhea. This finding coincides with previously reported cases from Turkey in terms of the seasonal emergence, and suggests a possible common source other than the water supply systems of the city. A highly probable source could be insufficiently washed food, especially green salads. The patient histories revealed a high percentage of green salad consumption (16/17; 94%) in different restaurants, but no food or beverages were available for testing when the investigation occurred. The mean age of the patients was 41.94 ± 9.99 (range: 28 and 59). There were no C*yclospora *infections detected in children. This strengthens the green salad hypothesis as this is not a favorite food of children.

PCR testing confirmed our diagnoses but is of limited value for routine diagnosis. The single-step PCR used here was not as sensitive as the conventional method and false negative PCR results might be due to inherent inhibitors, but is an initial step to further analyze the genome, and epidemiology of the parasite. Further investigations and reliable genomic fragments will be required to overcome the problems we encountered and to obtain data on the epidemiology of *Cyclospora cayetanensis*.

We could assume that perhaps a small outbreak occurred from an unknown source. There was clearly a waterless summer [14], and perhaps insufficient food washing as a result, but there remain some questions that need to be answered. Is C*yclospora cayetanensis *an endemic pathogen in Istanbul that is found easily in the soil? Do changing climate conditions favor its sporulation?

In conclusion, Istanbul has a very crowded population (15% of Turkey, living in suburbs) similar to other important cities around the world, and must be surveyed closely. Our data on C*yclospora *showed an unusual rise in the number of cases over a limited time period during a particular season, which must be considered. Notably, the cases were geographically separate and not clustered in a particular area.

There are other emerging infections such as Crimean-Congo hemorrhagic fever infections imported from countries where they are endemic that are finding new epidemiological niches (e.g. in central Turkey). Additional infections may be finding their niches due to the climate conditions described here.

## Competing interests

The authors declare that they have no competing interests.

## Authors' contributions

MO and ST contributed equally to this work; MO processed the samples and performed the majority of experiments; EH treated the patients, collected and provided patients data; ST designed the study and wrote the manuscript.
